# Users' Perception of Violence and Conflicts With Professionals in Primary Care Centers Before and During COVID-19. A Qualitative Study

**DOI:** 10.3389/fpubh.2021.810014

**Published:** 2021-12-16

**Authors:** David Pina, Paloma López-Ros, Aurelio Luna-Maldonado, Aurelio Luna Ruiz-Caballero, Bartolomé Llor-Esteban, Jose Antonio Ruiz-Hernández, Jesús Javier García-Jiménez, Esteban Puente-López, Begoña Martínez-Jarreta

**Affiliations:** ^1^Department of Socio-Sanitary Sciences, University of Murcia, Murcia, Spain; ^2^Applied Psychology Service (SEPA), University of Murcia, Murcia, Spain; ^3^Department of Behavioral Sciences and Health, University Miguel Hernández, Elche, Spain; ^4^Department of Nursing, University of Murcia, Murcia, Spain; ^5^Department of Social Psychology and Psychiatry, University of Murcia, Murcia, Spain; ^6^Department of Personality, Psychological Assessment and Treatment, University of Murcia, Murcia, Spain; ^7^Department of Pathological Anatomy, Forensic and Legal Medicine and Toxicology, University of Zaragoza, Zaragoza, Spain

**Keywords:** violence, Primary Care, users, healthcare workers, qualitative

## Abstract

**Background:** Workplace violence is a social problem of special interest in both intervention and research. Among the sectors that most perceive this type of violence, health care professionals stand out. The most common type of violence for this professional group is the one perpetrated by the users or patients themselves. It has been reported that one out of every four acts of violence in the workplace occurs in the healthcare setting. Within the health sector, the Mental Health, Emergency and Primary Care services have been widely reported as being among the most vulnerable, with Primary Care being the least addressed of the three. Although the available literature is extensive, there are hardly any studies that explore from a qualitative perspective what are the sources of conflict in this sector from the perspective of the users, the most common being to work with professionals.

**Objective:** The aim of this study is to examine those aspects derived from the organization, the professionals or the users of Primary Care that, from the users' point of view, cause violent situations and how they think these could be avoided.

**Method:** The sample consisted of 80 users of the Primary Care services of the Health Service of Murcia. For data collection, a qualitative study was conducted through 10 focus groups and a subsequent thematic analysis of the data.

**Results:** The results have allowed us to identify that, from an organizational point of view, the uncertainty in waiting times, the need to adapt the telematic or telephone appointment to the different types of users, or the management of emergencies in Primary Care are the aspects that cause most conflicts between users and professionals. In this sense, suggested improvements are aimed at providing information in the mobile application updated on the opening hours or maintaining the telephone appointment for those who need or request it, among many others. As for the professionals, users point out that the medical staff is perceived as distant and sometimes does not provide enough information on the health status of users. Another professional group widely addressed in the focus groups was the administrative staff, being described as lacking in communication skills, assertiveness, or empathy. Users recognize the existence of a demanding/aggressive profile among users, who makes instrumental use of violence to achieve privileges over users in general. We have also identified the profile of the user who makes use of Primary Care as a way of socializing or managing conflicts of a socioemotional nature. As proposals for this thematic block, users suggest group therapies, the use of audiovisual material complementary to the information provided by professionals or community interventions in psychoeducation.

**Conclusion:** This study allows to explore conflicts between users and professionals from the Primary Care patients' perspective. Our results are complementary to the available evidence that has used the professional's approach to study the phenomenon of workplace violence. The identification of sources of conflict and the assessment and contribution of users on possible ways of improvement can serve as a basis for the design of prevention and intervention plans to improve the work environment in Primary Care centers.

## Introduction

In recent decades, both at the level of care and research, there has been an increased interest and concern for the study of workplace violence (1). In 2016, the ILO (International Labor Organization), defined this type of violence as “any action, incident or behavior that departs from what is reasonable by which a person is assaulted, threatened, humiliated or injured by another, in the exercise of their professional activity or as a direct consequence of it” (2). The same document refers to two main types of violence: Physical and psychological violence.

International studies indicate that at least 25% of workplace violence occurs in the healthcare setting (2, 3). In this sector, professionals themselves may perceive violence as inherent to their job performance (4–7). A recent meta-analysis concluded that workplace violence is observed in all continents in practically the same proportion over the last 30 years, with prevalence ranging from 48.1% in Europe to 70.9% in Oceania (1, 6, 8). The magnitude of the problem has led international organizations such as WHO, ILO, ICD and IPSO to develop guidelines for creating violence-free healthcare institutions (9).

Workplace violence in the health sector can happen both between colleagues (lateral violence), as well as in hierarchical order or from superiors to other subordinate workers (horizontal violence) and from users to professionals, the latter being one of the most studied (10–12).

Although this type of violence is perceived by practically all health care workers, several studies have shown inequalities in the prevalence of workplace violence depending on the service where the professional belongs. In this sense, professionals in Emergency Services, Primary Care or Mental Health seem to be at greater risk of suffering violent episodes in the workplace. This study focuses on Primary Care services where it is estimated that at least 50.2% of the personnel are exposed to violence in the workplace (1, 5, 6, 8, 11).

Within Primary Care services, a recent study states that between 54.9 and 78% of the medical personnel are exposed to verbal aggression (13). These percentages are similar for both nursing and non-health care staff (e.g., clerks), the latter being among the most affected (1, 6, 8, 11, 14).

The exposure of these professionals to violence can have multiple consequences, mainly chronic stress, job dissatisfaction, absenteeism, organizational changes, or burnout (15, 16). Given the high personal and institutional cost of exposure to workplace violence in health professionals, it is necessary to investigate and find out what factors might be influencing, in order to act on them and minimize the risk. In general terms, the literature focuses on four main blocks: those related to the work context, those related to the staff, those related to the patient and, finally, those related to the system itself (17, 18).

As mentioned above, one of the main manifestations of workplace violence in the health sector is the one perpetrated by users against professionals. Therefore, knowing the perspective of the users themselves on the use of Primary Care and the treatment received by its workers is an interesting and necessary vision for understanding the phenomenon. Previous qualitative studies based on health care personnel have provided relevant information on the experiences, needs, feelings and perceptions of professionals. The importance of staff training, the need to inform the user, the fear of aggression, the lack of reporting and the management of conflictive situations have also been evidenced (7, 19–21).

From the user's perspective, it has been observed that when users feel that they are in a situation of vulnerability, the risk of aggression may increase. These situations can be perceived with relative ease by users who feel disrespect for their integrity (22, 23). These studies have been focused mainly from a quantitative perspective, and there is little scientific evidence of qualitative studies on users. Among these few previous works, we observe that, sometimes, the user has participated as part of the focus groups along with the staff or has been invited along with them. A precedent in the study of Primary Care using qualitative techniques with users is the work of Dois-Catellón and Bravo-Valenzuela (23). These authors state that a fundamental characteristic is the good treatment received by the professionals. They also describe that, in order to establish a good relationship, it is necessary to have an initial greeting, eye contact, to use understandable language and to consider the users' opinions, among other measures.

It has been observed that, when users are part of the studies, they feel integrated when working together, the results are very positive, and they feel a greater attachment to the health system. These previous studies point to the need to expand the knowledge available on the perception of users in order to propose changes in the system with the aim of reducing violence in Primary Care centers (24, 25). It is important that this body of knowledge is built with both quantitative and qualitative studies, since the latter allow a greater understanding of the lived experience from the point of view of the participants, focusing on the meaning of the thoughts, attitudes, behaviors and practices conferred by them (26).

Nevertheless, with the declaration by the World Health Organization (WHO) in March 2020 of the SARS-CoV-2 or COVID-19 pandemic, important changes and health adaptations were made worldwide due to the great care pressure. Health systems, in general terms, directed a greater number of resources toward hospital care, which led to a lack of human and material resources for crisis management in primary care, with 132 community health centers and 1,152 local clinics being closed in Spain (27).

This study has three objectives: Firstly, it aims to explore those aspects related to the organization, professionals and users that could be potential sources of conflict. Secondly, it will explore the users' proposals and opinions on possible ways of reducing these sources of conflict. Finally, the perception and satisfaction of users with the changes in Primary Care since the emergence of COVID-19 will be explored in depth.

## Methods

### Theoretical Paradigm and Study Design

Violence by users toward health care personnel in Primary Care has been extensively studied from a quantitative approach (6, 25). Although this approach is necessary and particularly relevant, it sometimes offers a limited view of the phenomenon because it is based on constructed questionnaires. For this reason, the present study aims to evaluate conflicts between users and professionals in Primary Care from the qualitative paradigm. This approach facilitates to explore with a higher level of specificity, as it lacks a previous explicit framework to guide the obtaining of results. This facilitates the exploration of one's own experience and how it is communicated, which can facilitate deepening the meaning and understanding of the information. In addition, it avoids the response bias typical of self-reported questionnaires and collects information from multiple verbal and non-verbal channels, impulsive or unpremeditated responses (28, 29).

In the same vein, a qualitative study is proposed from grounded theory with a constructivist approach (30) by conducting focus groups (31). The research team developing this study is made up of professionals with extensive experience in health care, in research and in the publication of scientific articles on violence by users toward health care personnel and in conducting qualitative studies.

### Participant Recruitment

The study was carried out in southeastern Spain. Potentially eligible participants for the study were those who made regular use (several times a year) of Primary Care centers. The interviews were conducted between June 2021 and July 2021. A total of 100 users were invited, of whom 80 (63.7% female) finally participated. The mean age of the participants was 48.92 (*SD* = 14.95) and the age range was between 18 and 75 years. 100% were of Spanish nationality, most were in active employment (57.5%), married or in a domestic partnership (48.8%) and visited the Primary Care centers between zero times and once a month (85%). The participants were not required to have been involved in any incident of violence in a Primary Care center; however, 38.8% acknowledged having had a conflict in their assigned center and 71.3% acknowledged having witnessed a conflict between another user and a professional (Table 1).

**Table 1:** Sociodemographic and work-related variables.

**Variables**	***N***	**%**
**Sex**		
Man	29	36.3
Woman	51	63.7
**Nationality**		
Spanish	80	100
**Employment Status**		
Active	46	57.5
Retired	16	20
Sick note	2	2.5
Others	15	18.8
Lost	1	1.3
**Marital Status**		
Single	25	31.3
Married or cohabitating	39	48.8
Separated or divorced	12	15
Others	4	5
**Frequency of visits to the primary care center per month**		
0–1	68	85
2–3	6	7.5
4 or more	6	7.5
**Have you ever had a conflict in your Primary Care center with a member of the staff?**		
Never	48	60
Sometimes	31	38.8
Lost	1	1.3
**Have you witnessed any conflict with a staff member at your Primary Care facility?**		
Never	23	28.7
Sometimes	57	71.3

### Procedure

The present study was approved by the Research Ethics Committee of the authors' home University (ID: 3555/2021) and conducted following the recommendations of the COREQ guide for focus groups (32). For sample recruitment, snowball sampling was used among users to whom the research staff had access. In all cases, an information sheet was provided along with a verbal explanation of the objectives of the study. Before conducting the focus groups, an informed consent form was provided and completed by 100% of the participants. It provided explicit acceptance of participation and for the audio recording of the focus groups.

Due to the problems derived from COVID-19, there were two alternatives for conducting the focus groups. On the one hand, face-to-face focus groups were conducted in a large, ventilated room that allowed compliance with the sanitary measures imposed at the time they were conducted. On the other hand, for those users who showed their rejection to a physical social meeting (due to COVID-19 restrictions), the focus group was conducted by video call. With both options, a total of 10 focus groups were conducted with a duration between 60 and 70 min and with approximately 8 participants per group. Before conducting the focus groups, users were again asked for their verbal consent before starting the audio recording. In addition, they were reminded that the audio recording would be destroyed after its transcription. In the text file, any data provided that could identify the participant or another person was replaced by a code.

The inclusion criteria used for the selection of participants were: (a) being a user of Public Health Primary Care and (b) being over 18 years old. The exclusion criteria were: (a) being mainly users of Private Health care, (b) not signing the informed consent form, and (c) having some type of disability that impedes comprehension or verbal expression.

### Data Collection

Focus groups were chosen because of their wide use in research. For data collection, a script was designed based on previous studies and the professional experience of the researchers and other collaborators. The script was complemented with concept mapping in a group of experts and interviews with key informants. Finally, the script was tested with a group of users not included in the results of this study (33). For the design and development of the groups, the recommendations of Krueger (31) were followed, grouping people with common characteristics related to the topic or research question (Primary Care users).

The focus groups were conducted by the first and second authors with the assistance of the rest of the authors, with no one else present in the room except the interviewees. The authors have demonstrable training and experience in creating and extracting data through focus groups, having applied this methodology in several of their studies. Before starting the recording, they spent between 5 and 10 min establishing rapport with the attendees through trivial questions. After this, they were told about the functions, objectives and importance of studying violence toward health care workers. The interviewers adopted a neutral attitude, free of bias, assumptions, or interest in the results. The rest of the authors took turns to support during the different focus groups with the same neutral attitude, taking notes and complementing the interviews.

The interviewees were encouraged to provide all the information they knew, whether their own or from someone they knew, avoiding focusing exclusively on their own experiences.

### Content of the Interview

All the participants were asked questions related to violent situations in Primary Care centers. These questions were divided into three blocks. First, sources of conflict related to the Primary Care organization or system were explored. Secondly, conflicts involving the professionals themselves were explored. Finally, they were asked about conflicts generated by the users themselves. In all cases, aspects that could improve coexistence and possible solutions to the conflicts raised were also explored. In addition, the users' perception of the changes experienced in Primary Care since the emergence of COVID-19 was explored.

### Data Analysis

The data were analyzed with an inductive and constructionist approach through thematic analysis following Braun and Clarke's proposal (34). This method allows the data to be analyzed in six phases. In the first phase, familiarization is performed through the transcription of the group recordings. In this phase, the researchers take notes and mark ideas that can help in later phases.

In the second phase, initial codes are generated and discussed in pairs. These codes are generated using an inductive or bottom-up approach to identify the data, without attempting to fit them into a pre-existing theoretical framework. When there was no consensus, multiple coding was proposed.

In the third phase, topics and sub-topics were proposed by grouping these codes and elaborating maps and tables. For the generation of themes, latent themes were explored using a constructivist perspective, avoiding relying on a simple description of the data. In the fourth phase, the different researchers review and discuss the generated themes. Finally, in the last two phases, the themes were definitively defined and named, discussing data saturation and producing a report.

## Results

**Figure 1:**
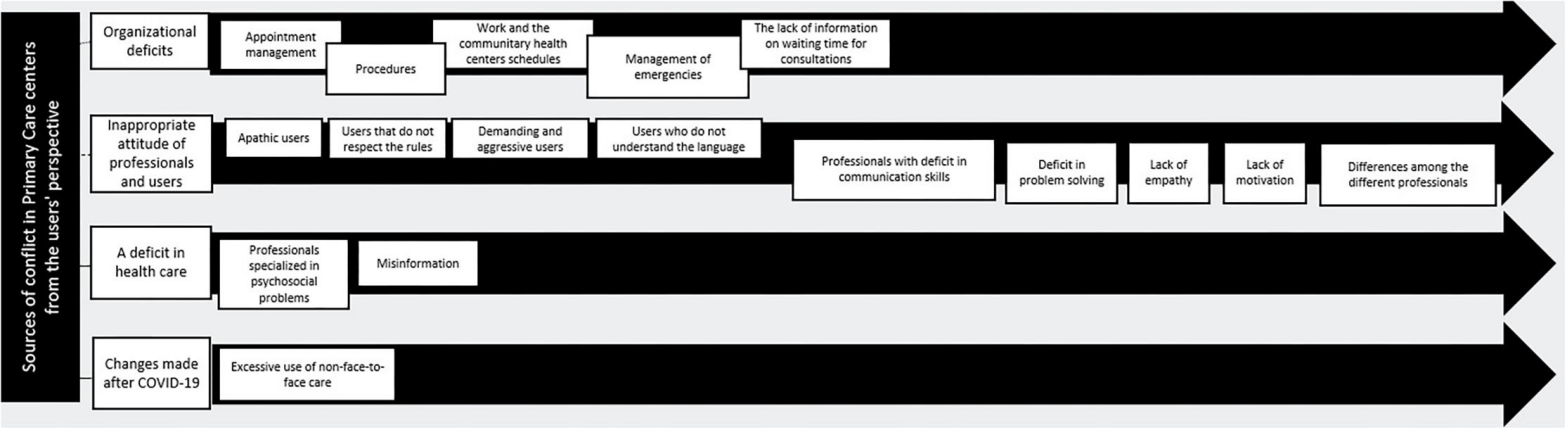
Sources of conflict in Primary Care centers from the users' perspective: topics and subtopics.

**Figure 2:**
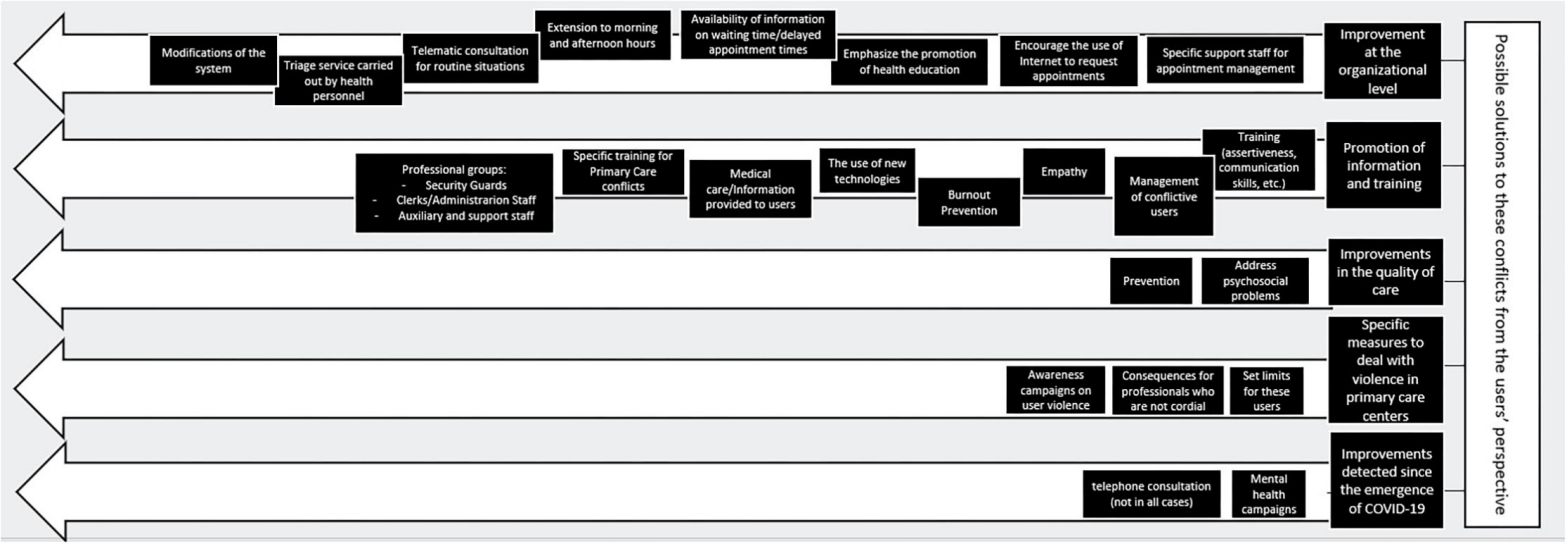
Possible solutions to the conflicts in Primary Care centers from the users' perspective: topics and subtopics.

From the thematic analysis of the focus groups, 4 interrelated thematic blocks have been observed that identify sources of conflict in Primary Care centers from the users' perspective. These are (1.a) Conflicts generated by organizational deficits, (1.b) Conflicts generated by an inappropriate attitude of professionals and users, 1.c) Conflicts derived from a deficit in health care and (1.d) Conflicts derived from changes made after COVID-19 (Figure 1). In addition, 5 other thematic blocks have been identified that encompass possible solutions to these conflicts according to the users themselves. These are (2.a) Proposals for improvement at the organizational level, (2.b) Promotion of information and training, (2.c) Improvements in the quality of care, (2.d) Specific measures to deal with violence in primary care centers, and (2.e) Improvements detected since the emergence of COVID-19 (Figure 2).

### Conflicts Generated by Organizational Deficits

All the focus groups stated that one of the main sources of conflict that leads to violence in Primary Care centers is the system's own organizational problems. In this regard, appointment management is a recurring topic, generating feelings of frustration or anger in users. Some of these problems seem to be shared by all the centers, especially four of these problems. In the first place, the management of medical appointments by telephone, with users reporting great difficulties to be attended. Online appointments do not seem to be a suitable alternative for all users, as it is difficult for both low-income and elderly users to use this method. Regardless of the method used, users consider that a major source of annoyance and frustration is the delay between the request and the consultation with the health professional.

“*I believe they should improve the telephone service because, for example, for people who don't know how to make an appointment online because they are elderly or whatever, they don't have any other choice but to go there. Because it's difficult for the people at the community health center to pick up the phone”*

“*It's really hard for an elderly person to make an appointment over the phone. You are not allowed to go to the [community health] center, also now because of the COVID situation. That already makes the person go with a predisposition of anger towards the community health center, towards their doctor. Then, when they get there, they are given an hour [for the appointment] and that hour is never met, so they arrive with a little bit more anger”*

“*I have asked for an appointment in ten days and they [the community health center] give you an appointment in 15 days. Any type of consult, minimum 15 days”*

Apart from requesting an appointment, users state that they encounter other difficulties when they try to make arrangements at the Primary Care center. In this regard, two frustrating procedures for the user have been pointed out. On the one hand, there is a need to carry out certain formalities in person when these could be done online (e.g., requesting registration, changing information on the health card or absence notes); and on the other hand, the difficulties in formalizing complaints and claims at the centers, as can be seen in the following extract:

“*About the grievances, I also feel like they are not taken into account […] when you take other types of actions, such as a formal complaint, the problem is that there are no witnesses, because you enter [the doctor's office] alone, and it's their word against yours, so, if the attention has not been appropriate, it is very difficult to prove the feeling. They usually hide behind the fact that the technical treatment is correct*”

The reconciliation of work and health care in Primary Care is sometimes a source of conflict for users. In this line, the incompatibility of the working day and the attendance schedule has caused problems for users, which can lead to conflicts in the centers. In addition, the management of sick leave is described as a potential source of conflict when it is not granted and the user does not agree with this decision, as shown in the following dialogue:

“*The doctor was never at their office when it was their turn. So, it makes me angry because, as I must be at work at a certain hour, they are supposed to be at a certain hour too. Therefore, that makes you upset, as then you don't even have time to do what you were going to do because now you are in a hurry. That makes everybody upset and it gets worse and worse, especially people who have things to do, who have their work schedule*”

It has been observed that the management of emergencies in Primary Care is perceived as a source of conflict and violence by almost all the participants in the study. In this regard, they criticize the fact that triage in this service is carried out by non-health administration staff, in a context of low privacy or lack of confidentiality, which generates multiple conflicts and generalized frustration, as shown in the following example: “*Triage cannot be done by a clerk, and people should not talk about these things at the counter*”. Furthermore, they claim that this emergency service is collapsed due to a lack of control, which causes its users to take advantage of the situation by abusing it in order to avoid the wait between the appointment request and the consultation.

“*There are many people who continually need to go to the doctor and that is why they abuse the emergency room, but that will always happen and has always happened, especially in adults- sorry, adults or kids, hysterical parents who take their children to the doctor a lot*”

“*I go directly to the emergency room and that way they doctor sees me and I get everything done, all at once, because otherwise I have to go to the doctor, then they have to order to run some tests, which takes a lot of time, then etcetera, etcetera, so I get all that done at once and that's it*”

Finally, in this block, the lack of information on waiting time for consultations is particularly relevant. In this regard, users report that, despite having an appointment at a specific hour, they often do not see the doctor at that time and are not informed of the additional waiting time. A variant of this also happens in telematic consultations, with no certainty as to when the call will be received. In addition, once they have been attended, they state that the consultation time is excessively short, so they feel that they are not attended and informed with sufficient quality.

“*I was surprised, because at the family doctor, every time that I go, it takes me 1 hour, hour and a half and even 2 hours to wait for the doctor to see me*”

“*My mother has been booking doctor appointments over the phone for the last few months and the doctor assigned her, for instance, at twelve thirty, but then called her at two thirty. In other words, the call schedule was not respected either. They give you an appointment, but, in the end, the doctor would call you when they could. That was not respected either*”

“*In the end, they stick to the time they have to attend you, if in the end I saw... I think that's it... it's the time they have to attend you because I don't think any doctor wants to attend you in 5 minutes as some do, some do it in more time and that's why you have to wait two hours*”

### Conflicts Generated by an Inappropriate Behavior of Professionals or Users

The user-professional relationship is perceived by the participants as one of the most determining aspects in the emergence or not of violent situations in Primary Care centers. In this line, sometimes the climate of the centers can be perceived as “unpleasant” and this can be triggered both by the users and by the professionals. Regarding users, apathy, patients who do not respect the rules, demanding, aggressive and users who do not understand the language well have been identified as the main sources of conflict in this regard.

“*And the user can have an infinite patience, a wonderful character and know how to manage conflicts, but when it is continuous, when you see one injustice after another, in cases that are medically speaking quite difficult, the patient finally explodes because they are not giving you the minimum treatment that a patient needs”*

“*There is a type of patient who, when they go to the emergency room or their community health center, thinks that they are the first one to be seen, that they decide on the medication and the tests that are going to be run”*

“*I have seen in the waiting room people who wanted to enter whenever they wanted, people who don't allow the professional to fail, all this kind of stuff”*

“*And the problem is that we live in an infantilized society, that is, I have the right to everything. And if not, in the end, we will get the ‘I pay your salary' ”*.

On the part of the professionals, the interviewees point out a deficit in communication skills, problem solving, empathy and a certain lack of motivation. In addition, they point out that there are differences among the different professionals, especially among administrative, nursing and medical staff, as can be seen in the following examples:

“*Another thing I see is the simple fact that, just because they wear a white coat, they have authority* […] *And then they acquire that, that paternalistic tone, that you can't talk, if it's not what they tell you, there is no possible alternative* ”

“*Doctors go with little time, but sometimes saying a kind word, a word that makes you feel good and that helps you, helps them to tell you something harder, to have a little more empathy towards the other person, I believe that all of that is very important. The people at the counter are not empathetic at all to people who come with a problem*”

“*Sometimes it's also the security guards who cause those difficult situations”*

### Conflicts Arising From a Health Care Deficit

The participants in the focus groups exemplified various deficits in the health care offered by Primary Care services. Among all of them, there is a need to incorporate professionals specialized in psychosocial problems, since many users make use of Primary Care for these reasons (feelings of loneliness, work problems, etc.) and, generally, the only alternative proposed to them is medicalization.

“*There is no way to make an appointment with a psychologist at Primary Care, it takes 7 years to have an appointment, the issue of fundamental psychosocial support”*.

“*There are also people who have mental health problems and maybe the solution is not pharmacology but another type of approach.”*

“*Social workers are necessary because they give you advice, they provide different care in comparison to doctors or nurses”*.

On the other hand, a general feeling of misinformation has been observed. The users interviewed think that they are not given enough information about waiting times for consultation or their therapeutic process.

“*I believe that the information phase or the perception of lack of information on the user side can generate anger, a feeling that something is happening and you are missing something, dissatisfaction”*

“*what we lack is that the professional, the practitioner in the exercise of their profession, apart from humanity, which is what has been claimed, what they have to be clear about is that their obligation in this case is to inform the patient and their family, if there is one, of what therapeutic options the patient has, according to the patient's age, the consequences of using certain options or not, and to respect the patient's right, in this case, to decide whether they want to accept them or not, due to their age and the consequences that the therapeutic options may have”*.

### Conflicts Arising From Changes Made After COVID-19

Finally, some of the conflicts observed by the participants in this study are specifically derived from the changes experienced in Primary Care since the emergence of COVID-19. In general terms, users consider that the system has not adapted properly. An excessive use of non-face-to-face care has been pointed out, generating a feeling of insufficient attention.

“*I think they have also accommodated themselves, because my mother for example, she had a wound on her foot, and I said ‘mom, go to the doctor', and she said, ‘but they only give me an appointment by phone”*.

“*There are people who need face-to-face consultation and they are not being given it, because of this.... Because of the existence of the telephone and telematic consultation, and they really need a face-to-face consultation”*.

### Proposals for Improvement at the Organizational Level

In the focus groups conducted, alternatives were also proposed that users consider appropriate to solve some of the conflicts mentioned above. In this sense, they propose incorporating specific support staff for appointment management, unloading the administration service. In addition, they propose to encourage the use of Internet to request appointments and extending this to other services but bearing in mind that some users (especially the elderly) have difficulties, carrying out campaigns in this regard. Finally, they emphasize the promotion of health education with the aim of reducing the request for appointments through self-care.

“*I see it as very comfortable, because you can take it out, you can choose the day, you can do it whenever you want and I think it would be good too, even if it were to be extended for example, when you are referred for a cytology or an X-ray or something like that, in a case where you do have to go to the counter and have to queue a lot; it would also be interesting if it could be done online”*.

“*The online appointment was present way before COVID, but, well, I think it's alright. As long as everyone has means, because a lot of people don't have one. So, you have to have the phone, or whatever. An alternative for the grandpa who doesn't handle the smartphone”*.

Regarding the consultation itself, users think that information about the waiting time/delay in the schedule of their appointment should be available, either through the application, a message or in the waiting room itself, as well as the extension to morning and afternoon hours. They think that telematic consultation should be encouraged, especially for routine situations (administrative formalities) or less serious procedures. In-person appointments are essential according to users but should be reserved for consultations where it is really necessary. Along the same lines, they praise the use of electronic prescriptions and recommend training and promoting their use.

“*It is an incredible thing and, yes, the truth is that if it sends you a message, ‘hey, you won't enter until two hours from now', for example, it would be great, it would be great”*.

“*It is regulated by the workers' statute. Every X hours, there are fifteen minutes of rest, I don't know if it is regulated or not in the medical area, but I don't think it's far-fetched”*.

“*What I have loved is the telephonic consult, for things that are not very urgent, you talk to the doctor over the phone and they prescribe what you need over the phone and it is alright”*.

The users interviewed consider that a necessary solution to the conflicts mentioned in Primary Care emergencies would be to incorporate a triage service carried out by health personnel.

“*No, obviously that is not the clerks' job from my point of view. In other words, not even nursing staff, who have more knowledge, an effective triage would logically be medical”*.

Finally, in this block the users propose some modifications of the system. They consider that it is necessary to improve the means of communication with senior management, increase human resources, that the professionals' schedules of attention and rest are public, and to include health care in support groups aimed at users with the same pathology.

“*I think the problem also lies in the schedules of the community health center, if it only opens during morning hours, there are people who can't miss work and will go to the emergency room in the afternoon”*.

“*I believe that the more controlled the whole system is, the more standardized it is and the more evaluation is done”*.

“*that they encourage groups for these people... a couple of days a week they have some sessions where they are taught to breathe, they are taught to relax, they are taught to calm down. Because I think it's very complicated that you can do it home alone without having done that before”*.

### Promotion of Information and Training

The attitude of the professional toward the user has been previously mentioned as a source of conflict. Along these lines, the interviewees consider that training should be provided to professionals in patient care (assertiveness, communication skills, etc.), management of conflictive users, empathy and burnout prevention. In addition, they consider it necessary to train in the use of new technologies available in the service and the improvement of medical care/information provided to the user.

“*So if you get a [female] nurse or a [female] doctor who is more empathetic by nature, then you are lucky, and if not then, you aren't, when it is something, all these types of competencies and skills can be easily developed by including them in training and, kind of continuous because, due to the fact that health professionals are constantly seeing health problems and seeing patients, they generate a kind of layer in which they distance themselves from seeing the person as a person.”*

“*I am an administrative in the autonomous community and I take courses to improve, recycle and everything, so they should also take courses to learn how to treat people better”*.

“*then we will also have to take courses from above to work on emotional intelligence and empathy, and to reduce burnout syndrome, which is the syndrome of being burned out, which professionals, especially in nursing, have a very high level of burnout”*.

By professional groups, the interviewees consider that security guards should have specific training for Primary Care conflicts. This type of personnel is well perceived by users, but sometimes it can increase the tension of conflicts because they do not have the necessary skills, as shown in the following example: “*I am not saying that the figure should not be there, but maybe I think that they should be people who are... well trained, who have psychological evaluations and then quality reports on treatment. They should also have some supervision*”. This training deficit has also been pointed out for the administration staff, who are considered not to have the necessary skills for some of their current functions, such as emergency management. Finally, they request auxiliary and support staff. These staff could help elderly people to request telematic appointments or orient people within the center, among other functions.

“*Yes, for example, now, with the COVID issue, I have seen it improve with the person who is at the door. For example, in my community health center now there is a person who, when you arrive at the door, asks you what you need and says, “Look, your office is in this place, you can have your tests done here, if you need a bottle, I will give it to you”*.

### Proposal for Improving Quality of Care

In order to improve the quality of care, the participants consider that it is necessary for Primary Care centers to address psychosocial problems. For this, they request both the incorporation of specialized personnel and the creation of support groups. These support groups should be focused on reducing the medicalization of some pathologies, according to the interviewees. Some of the proposed groups were oriented to relaxation techniques, self-help, yoga or people with feelings of loneliness.

“*I would love a support group. There are many things that really don't need consulting, they need support and you go, you go to the doctor, to the doctor, to the doctor because you feel very lonely, very misunderstood.”*

In general terms, users consider that the focus should be shifted to prevention, which could result in less demand for the service and less conflict. They request that, when treatment is necessary, it should be offered with other alternatives, whenever it is possible, requesting that the existing paradigm expands to a more integrative medicine and that it take into account the variety of health professionals currently available.

“*what we lack is that the professional, the practitioner in the exercise of their profession, apart from humanity, which is what has been claimed, what they have to be clear about is that their obligation in this case is to inform the patient and their family, if there is one, of what therapeutic options the patient has, according to the patient's age, the consequences of using certain options or not, and to respect the patient's right, in this case, to decide whether they want to accept them or not, due to their age and the consequences that the therapeutic options may have”*.

### Specific Measures to Deal With Violence in Primary Care Centers

Specifically, for cases of violence, the interviewees have pointed out various measures that they consider “not very effective” and that, in their opinion, should not be implemented. In this line, with regard to the conflictive user, they consider that they should not be punished, nor should the referral professional be changed, as this would not be a real solution to the problem. As an alternative, they propose that the system should set limits for these users, preventing them from benefiting from their own violent behavior by being able to make “attention calls” without the need to punish.

“*I believe that the person in need should be attended, and that the system should be restructured so that each person has the attention they need, and do not have to use the emergency room because they are not attended in PC. I don't think anyone should be punished; I don't think punishment is a good alternative.”*

Just as measures against conflictive users are proposed, the interviewees consider that professionals should have consequences when they do not play a cordial role with the user. In this line they speak of implementing user satisfaction measures by making the results obtained available to the professional.

“*when I have left these consultations, I wondered why they aren't evaluated through the assessment of their patients' satisfaction. Ask for our degree of satisfaction, how we feel, if we feel good after having received care, in some way to measure and be able to improve their work or at least put it on trial. The feeling I get is that they are a little accommodating.”*

Users also speak of the need to implement awareness campaigns on user violence, but these should go beyond signage which, at times, is ineffective on its own. Along with this, they propose the unification of criteria before acting in these cases with the publication of an action protocol for conflicting patients.

“*The dissemination and awareness campaigns are not very well thought out, i.e. the leaflets are not read by anyone, the posters on the walls are not read by anyone, I think it has more to do with culture and that at a social level is very much like in the long term”*.

### Improvements Detected Since the Appearance of COVID-19

Finally, the focus groups pointed out the importance of improving some aspects that have not been entirely positive in Primary Care since the appearance of COVID-19. Users consider that mental health campaigns are not being carried out by the centers:

“*Mental health at the Primary Care level is the great forgotten, even during COVID. And when you ask for an appointment, they give you an appointment for September-October when a lot of months have already passed. And mental health is also important. Here I go. There are also people who have problems of that type and maybe that would be the solution, not the pharmacological solution but another type of solution or a different approach”*.

In addition, they believe that the telephone consultation, although it has been an advance for many things, should remain, but not in all cases. In this sense, they speak of the need for the elderly or people with special needs to be attended in person, stressing the need to offer one or the other depending on the type of user.

“*Over the phone, I do not consider that the doctor can... Some of them can. But there are others that do not. Well... in the field, I don't know, now as in Primary Care almost everything is treated, in general, I think that there are some appointments that should not be by telephone, that should be face-to-face”*.

“*There should be a filter so that you can call the doctor and then the doctor will tell you ‘okay, come tomorrow' or ‘come in an hour'”*.

## Discussion

In response to the objectives set out, the users of primary care services consider that the workplace violence perceived in these services is due to organizational deficits, problems with the attitudes of professionals and users, and deficits in the health care received. In order to improve the work environment, these users consider that improvements should be made at the organizational level, promote information and training of users and professionals should be promoted, the quality of care should be improved and specific measures should be proposed to deal with violence in Primary Care centers. Finally, both positive changes were detected in Primary Care since the emergence of COVID-19 and negative changes that worsened the climate in these centers. These results provide evidence of situations that the user is able to identify and perceive that could act as triggers for user-professional aggression in Primary Care. Some of the focal points identified seem to be in line with recent studies carried out within Primary Care from both the user's perspective (23, 24, 35) and the professionals' perspective (6, 36, 37). It should be noted that some of these sources of conflict do not seem to be specific to Primary Care, being present in other fields of health care (11, 20, 38, 39).

Specifically, from the organizational perspective of Primary Care, it was observed that delays in care or in the management of appointments generate great discomfort in the user. This has been previously reported in the literature (11, 35, 38). In this line, authors such as Raveel and Schoenmakers (13) propose increasing the information available to users, this proposal being in line with what was observed in the present study. Along the same lines, users consider that the use of telematics should be strengthened for those issues that do not require face-to-face attendance, in an optional manner, so as not to disadvantage any group. The latter could be useful in reducing the discomfort of users who work, who sometimes find it difficult to reconcile work and health care, as observed in our results. The availability of flexible schedules in care centers could alleviate this problem (13, 20).

In this line, previous studies have reported that, sometimes, users can use emergency services to receive health care without losing work activity, thus not presenting an urgent health problem (20, 35). Users recognize that inappropriate use of the emergency department is one of the main triggers of conflicts in the centers. This seems to be caused by inadequate or non-existent application of health triage (23). The alternatives proposed by the interviewees reflect the need to create specific spaces for health triage and the availability of health personnel (as opposed to the administration personnel, who in some cases currently perform this function). This type of measure can facilitate the user experience, respecting their privacy and avoiding a collapse of the consultations for users who do not really have an urgent problem (37, 40, 41).

Relieving the care burden of Primary Care professionals in order to improve the quality of care seems to be a very important point for users. Finding a balance between reducing the burden of care and improving patient care, understood as an increase in time spent, does not seem easy to achieve. However, in this study, interesting alternatives have been observed in this regard. Therefore, users consider it important to reinforce user information channels and to promote user education in order to establish the criteria for appropriate use of the system (20, 42). In addition, the creation of support groups accessible to the population would provide alternatives to some of the sources of conflict mentioned so far. This proposal is shared both by the respondents and by previous studies in other healthcare settings with good results on health, reduction of violence and good user acceptance (24, 25, 43). Support groups are proposed within Primary Care to inform the population about the use of the system, about certain frequent pathologies (diabetes, hypertension, smoking...) and even about psychosocial problems. The users thus propose a way of reducing waiting times, improving communication between the user and the center, providing more information on how the system works and improving health education.

A large part of the information provided by the participants in this study refers to these communication deficits in user care. The main focus of these comments is the users' perception of an inadequate attitude on the part of the professionals. They refer to the lack of an initial greeting, depersonalized treatment, lack of collaboration to resolve conflicts, little eye contact, the feeling that they are not listened to attentively, excessively technical language or lack of detailed information on the therapeutic process. These and other aspects have been previously pointed out in the literature, concluding that empathy, assertiveness, friendly language, courtesy and cordiality on the part of the professionals are actions capable of preventing and reducing conflicts (20, 23, 24, 35, 44, 45).

The aforementioned information deficits are associated with users' perception of a paternalistic attitude of certain professionals. This paternalistic attitude seems to be defined by a lack of medical information received, lack of listening and little user participation in their own treatment decisions. The changes demanded by the users in our study are in line with those found in other studies in which community health center personnel are asked to change their professional attitude to allow the user to be an active part of his or her own health (19, 46, 47).

Specifically, in Primary Care, López-García et al. (6) claimed that professional training in these aspects is associated with a lower frequency of violence by users toward health care personnel. In our opinion, specific and continuous training of professionals in these issues as part of their work dynamics seems to be the appropriate way to favor the user-professional relationship and could be an issue to be dealt with more intensively from the basic training in the faculties. Many previous studies have shown that this type of training is an important factor in improving the work environment in the healthcare sector. The proposals made include training guided by professional psychologists, practical training with patient simulation, empowerment techniques, communication and empathy techniques and burnout prevention, among others (19, 20, 48–51). From our results, we also extract a proposal that aims to implement user satisfaction measures. These measures could be an appropriate complement to the training of professionals as they provide direct feedback on their treatment of patients and even on the perception of their professional competence.

The deficits in the care received do not only refer to the treatment provided by the health professional. Our results show the need for care with a biopsychosocial (more person-centered) approach. Users consider that professionals resort to medicalization due to the lack of other resources that can cover the needs for psychosocial problems, either due to lack of training, lack of specialized personnel or lack of time in the consultation rooms. Similar perceptions have been observed in previous studies (36, 52). It has also been indicated that this same idea is shared by professionals, pointing out the need to provide care focused on the user in a comprehensive manner, through a multi-causal approach, with a biopsychosocial perspective, and paying attention to their family conditions (23).

According to our results, users consider that medicalization can generate dependence, and therefore, the system would require a paradigm shift in which resources are invested and alternatives are provided to users so that they can become involved in their own therapeutic process. Thus, one proposal of the participants in this study is the implementation of support groups for psychosocial problems. According to the users, these groups could facilitate emotional management strategies, psychoeducation and health promotion. Evidence shows that coordinated teams in Primary Care in Prevention and Health Promotion avoid numbers of emergency hospital admissions through a focused approach to preventive health care and improve quality of life (36, 53, 54). This proposal should be developed by professionals trained in these aspects, such as psychologists and social workers. Complementarily, users consider that this type of care offer, in addition to alleviating some care deficits, would alleviate waiting lists, the demand of certain types of users or care dependence, among other aspects.

The problems derived from user-professional interaction can also be triggered by the users themselves. The participants in this study refer to demanding or very demanding users as the main cause. The literature points out that this type of users could be the consequence of high levels of stress, deficits in communication skills, advanced age, believing to be the only consumer of the service, family or personal problems, difficulty in following the rules or with severe psychopathology (19, 20, 24, 35). The main problem with these users is that they cause discomfort and frustration in other users. Sometimes, the demanding user is granted what he/she demands to alleviate the situation, but this is perceived by the rest as a favored treatment, thus facilitating the generalization of these behaviors to other users. In this sense, and in accordance with the literature, it is proposed that a culture of “zero tolerance” be created, with the creation of strictly enforced rules and protocols to guide the actions of health care and non-health care personnel (55–57). However, the participants consider that this type of inappropriate actions should not entail punishments such as fines or changes of professional or center. Raveel and Schoenmakers (13) conclude in their systematic review that measures to restrict access to users with a violent history are not a measure with scientific evidence for the reduction of workplace violence. In this regard, it seems that the proposals that have more support are the implementation of clear limits for patients and professionals and the promotion of violence awareness campaigns in Primary Care centers (13, 19, 58). The creation of protocols and the training of professionals in them has been proposed as a necessary violence reduction measure (13, 35, 59). However, no previous studies evaluating the effectiveness of these protocols and programs have been found.

Another noteworthy aspect of the results of the present study has to do with some of the adaptations made in primary care since the appearance of COVID-19. The lack of face-to-face consultations was undoubtedly one of the most recurrent comments. The user describes the insufficient and impersonal treatment, especially for pathologies not related to COVID-19. They state that they have mostly received assistance via telephone without the option of face-to-face assistance. In spite of this, the opinions regarding health care by telephone are not conclusive, since certain users support telephone consultation because of its convenience and above all for routine procedures, since it speeds up the system and reduces waiting times. No previous studies have been found that evaluate this type of aspect in primary care. As a general conclusion, it seems that users are satisfied with telephone consultations as long as they are given the option of choosing between this and face-to-face consultations.

A variant of this type of consultation is telematic consultation through the “Patient Portal”. This is a personal online space where the user can access their diagnostic tests, clinical reports, manage appointments and have a follow-up without waiting or travel. This space existed prior to COVID-19 but there seemed to be a certain lack of knowledge. The promotion of this tool has been well received by the study participants, who consider that it is necessary to carry out information campaigns for its use both for the user and the professional.

Finally, users demand more campaigns to address the psychological discomfort they have suffered during the last few months, especially when they were confined. Epidemiological studies in this line point out that one third of the population needed care by mental health services, mainly for presenting anxiety disorders, post-traumatic stress and depression (60). In this line, users consider that this has not been sufficiently taken into account in the adaptation of Primary Care.

This study allows us to have a specific view of those aspects that can generate conflicts in Primary Care centers before and during the adaptations carried out for COVID-19. Violence by users toward health care personnel is a particularly relevant problem of concern to both professionals and users. Previous studies have focused mainly on the vision of health care personnel in the centers, especially in psychiatric centers and hospital settings (11, 38–40). Furthermore, we found few studies that, in addition to the relationship between health personnel and users, evaluate other professionals, such as security or administrative personnel. This is why the approach focused on Primary Care, taking into account all workers, from the perspective of the users is innovative and necessary for the approach of prevention/intervention plans aimed at improving the work environment of these centers.

## Study Limitations

The study is not exempt from the limitations characteristic of qualitative designs. The results should be interpreted as a description and categorization of what was reported by the participants, and it is not possible to make causal inferences between the variables studied. In addition, the generalization of results could be limited. On the one hand, although the healthcare system is similar in different territories, there may be differences in the management of Primary Care centers depending on their geographical location. However, the opinion of users could be shared since the study variables are, for the most part, general and equivalent aspects between different healthcare systems.

## Conclusions and Future Directions

On the other hand, the measures taken in response to COVID-19 were not similar at the global level. Thus, the authors of this report propose three types of studies. Firstly, studies similar to this work that would allow us to further deepen our understanding of the users' view of the different variables studied in other contexts, for example, other countries. Secondly, quantitative studies, if possible longitudinal, that allow us to know in depth the relationship of the variables studied here with the aim of proposing explanatory models. Finally, we propose the design of violence prevention programs in primary care centers, taking into account the contributions of this type of studies together with the evidence available in the literature, as well as the evaluation of their results once implemented.

## Data Availability Statement

The raw data supporting the conclusions of this article will be made available by the authors, without undue reservation.

## Ethics Statement

The studies involving human participants were reviewed and approved by the Research Ethics Committee of the University of Murcia (ID: 3555/2021). The patients/participants provided their written informed consent to participate in this study.

## Author Contributions

DP, AL-M, and JR-H: conceptualization. DP and PL-R: methodology, formal analysis, and investigation. EP-L, AL, BL-E, and JR-H: validation. DP, EP-L, and PL-R: writing—original draft preparation. AL-M, AL, BL-E, JR-H, JG-J, and BM-J: writing—review and editing. DP: funding acquisition. All authors have read and agreed to the published version of themanuscript.

## Conflict of Interest

The authors declare that the research was conducted in the absence of any commercial or financial relationships that could be construed as a potential conflict of interest.

## Publisher's Note

All claims expressed in this article are solely those of the authors and do not necessarily represent those of their affiliated organizations, or those of the publisher, the editors and the reviewers. Any product that may be evaluated in this article, or claim that may be made by its manufacturer, is not guaranteed or endorsed by the publisher.
